# Case report: Uncommon multiple metastases from occult breast cancer revealed by ^68^Ga-DOTATATE PET/CT

**DOI:** 10.3389/fonc.2023.1106890

**Published:** 2023-02-22

**Authors:** Tianyuan Hu, Rongqin Zhang, Bing Zhang, Shanzhen He, Lian Liu, Yan Zou, Junhao Huang, Bing Wang, Ping Hu, Zhanwen Zhang

**Affiliations:** ^1^ Department of Nuclear Medicine, The Sixth Affiliated Hospital, Sun Yat-sen University, Guangzhou, Guangdong, China; ^2^ Department of Nuclear Medicine, Sun Yat-Sen University Cancer Center, Guangzhou, Guangdong, China; ^3^ Department of Nuclear Medicine and Medical Imaging, The First Affiliated Hospital, Sun Yat-sen University, Guangzhou, Guangdong, China; ^4^ Department of Nuclear Medicine, Guangdong Provincial People’s Hospital, Guangzhou, Guangdong, China

**Keywords:** ^68^Ga-DOTATATE, ^18^F-FDG, PET/CT, occult breast cancer, lymph node metastases

## Abstract

Occult breast cancer is an uncommon type of breast cancer and its diagnosis is challenging. It is usually invisible on multiple imaging examines. Metastases to the rectum and inguinal lymph nodes from occult breast lobular cancer are even rarer. ^68^Ga-DOTA peptides can image neuroendocrine tumors by targeting specific somatostatin receptors. Besides, other tumors, including breast cancer, have been shown to express somatostatin receptors. In this case, we presented a 63-year-old woman who underwent both ^18^F-FDG and ^68^Ga-DOTATATE PET/CT due to a rectal polyp. An endoscopic excision biopsy confirmed metastatic carcinoma of suspected breast origin, but subsequent ultrasound and MRI showed no signs of malignancy in the breast and adnexa uteri. PET/CT showed obvious ^68^Ga-DOTATATE activity in bilateral axillary and right inguinal lymph nodes with mild ^18^F-FDG uptake. Final histopathology at the left axillary, right inguinal lymph nodes, and rectum indicated metastases from breast cancer while the origin remained radiologically occult. Additionally, one uterine fibroids was found with positive uptake of ^68^Ga-DOTATATE and negative uptake of ^18^F-FDG. This case suggested that ^68^Ga-DOTATAE PET/CT may be an effective supplement in diagnosing OBC lymph node metastases with mild ^18^F-FDG uptake, and it may provide a new technology for the clinical diagnosis of occult breast cancer.

## Introduction

Occult breast cancer (OBC) is described as an axillary metastatic carcinoma without detection of a primary breast lesion, which is uncommon (1). The incidence of occult breast cancer is reported as being 0.3-1% of all breast cancer patients (2–4). It is thought that OBC is secondary to microinvasive breast cancer (5). Therefore, an accurate imaging examination of OBC is crucial for clinical diagnosis.

Research on imaging examination for detecting the occult breast cancer has been ongoing. The American College of Radiology recommends the use of MRI in OBC patients who do not have evidence of a primary breast lesion on the traditional radiological examination like mammogram and ultrasound (6). In a comparative analysis of MRI in 2015, contrast-enhanced mammography had equivalent if not better sensitivity (100% vs 93%) than MRI in detecting breast cancers (7). ^18^F-FDG PET/CT has been used in occult breast cancer, however, there are few case reports about that. Meanwhile, the imaging examination described above remains limited.

In our case, we described one case of occult breast cancer with rectum metastases who underwent both ^18^F-FDG and ^68^Ga-DOTATATE PET/CT, and enlarged lymph nodes with obvious ^68^Ga-DOTATATE activity and mild ^18^F-FDG uptake were shown at bilateral axilla and right inguinal area. It suggested that ^68^Ga-DOTATAE PET/CT might be an effective method for the diagnosis of occult breast cancer.

## Case description

We present a case of a 63-year-old woman with a history of rectal polypectomy confirmed metastatic carcinoma for 2 months during a physical examination. Further immunohistochemical results showed that the estrogen receptor (ER), progesterone receptor (PR), and GATA Binding Protein 3 (GATA3) expression was positive, suggesting that the primary tumor may be from the female reproductive system, especially from the breast. However, the ultrasound and MRI showed no signs of malignancy in the breast and adnexa uteri, and multiple enlarged lymph nodes in the bilateral axilla and right inguinal area.

**Figure 1:**
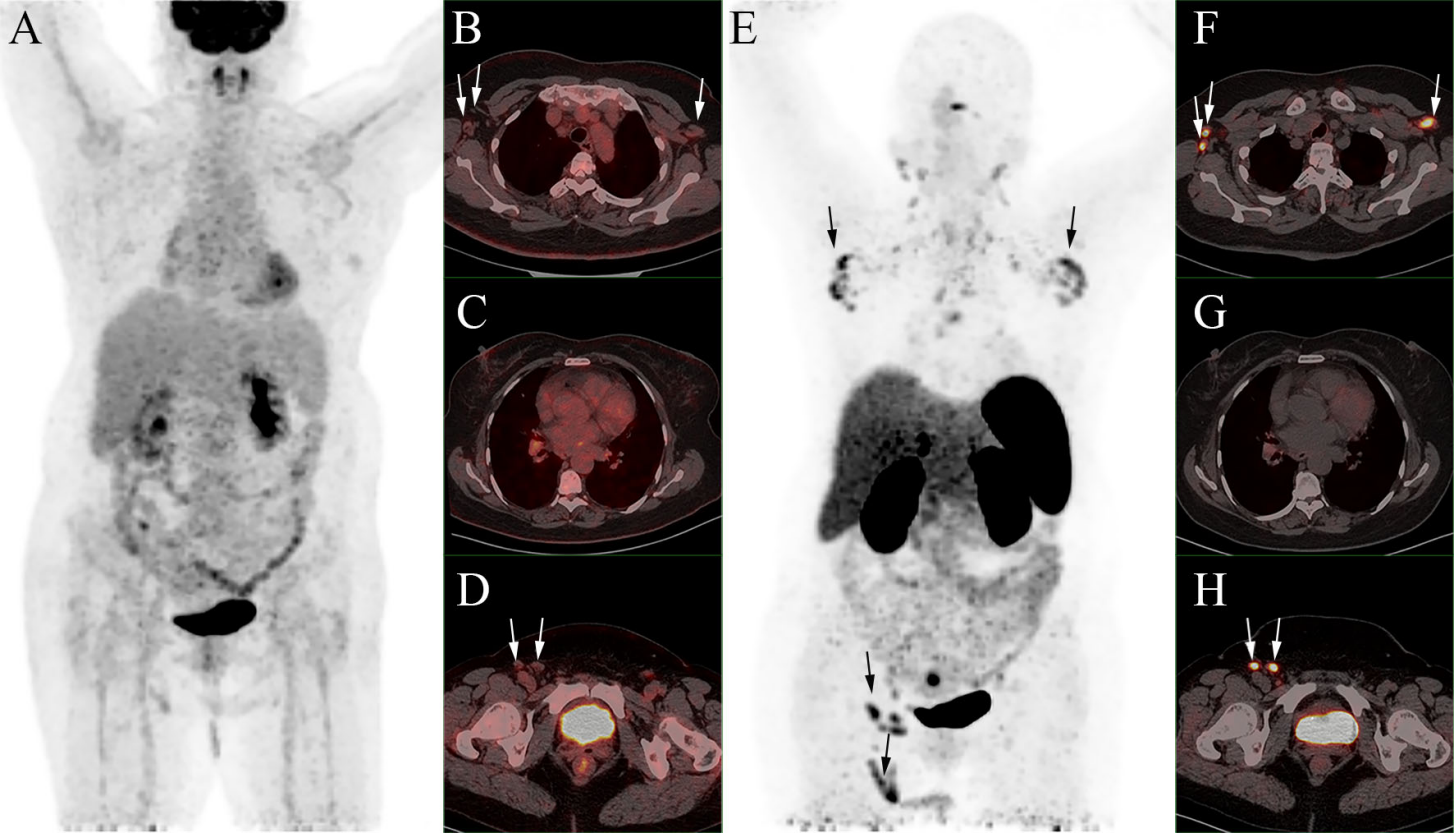
No abnormal activity was observed in the ^18^F-FDG MIP image **(A)**. Enlarged lymph nodes were found at bilateral axillary and right inguinal region with mild FDG avidity (SUVmax, 2.1) on ^18^F-FDG PET/CT (**B–D** fused PET/CT; white arrows) and no abnormality was seen in the bilateral breasts. In the ^68^Ga-DOTATATE MIP image **(E)** and the axial images (**F–H** fused PET/CT), higher ^68^Ga-DOTATATE uptake than ^18^F-FDG was observed in the enlarged bilateral axillary and right inguinal lymph nodes (SUVmax, 8.4; white arrows), and bilateral breasts were still negative.

**Figure 2:**
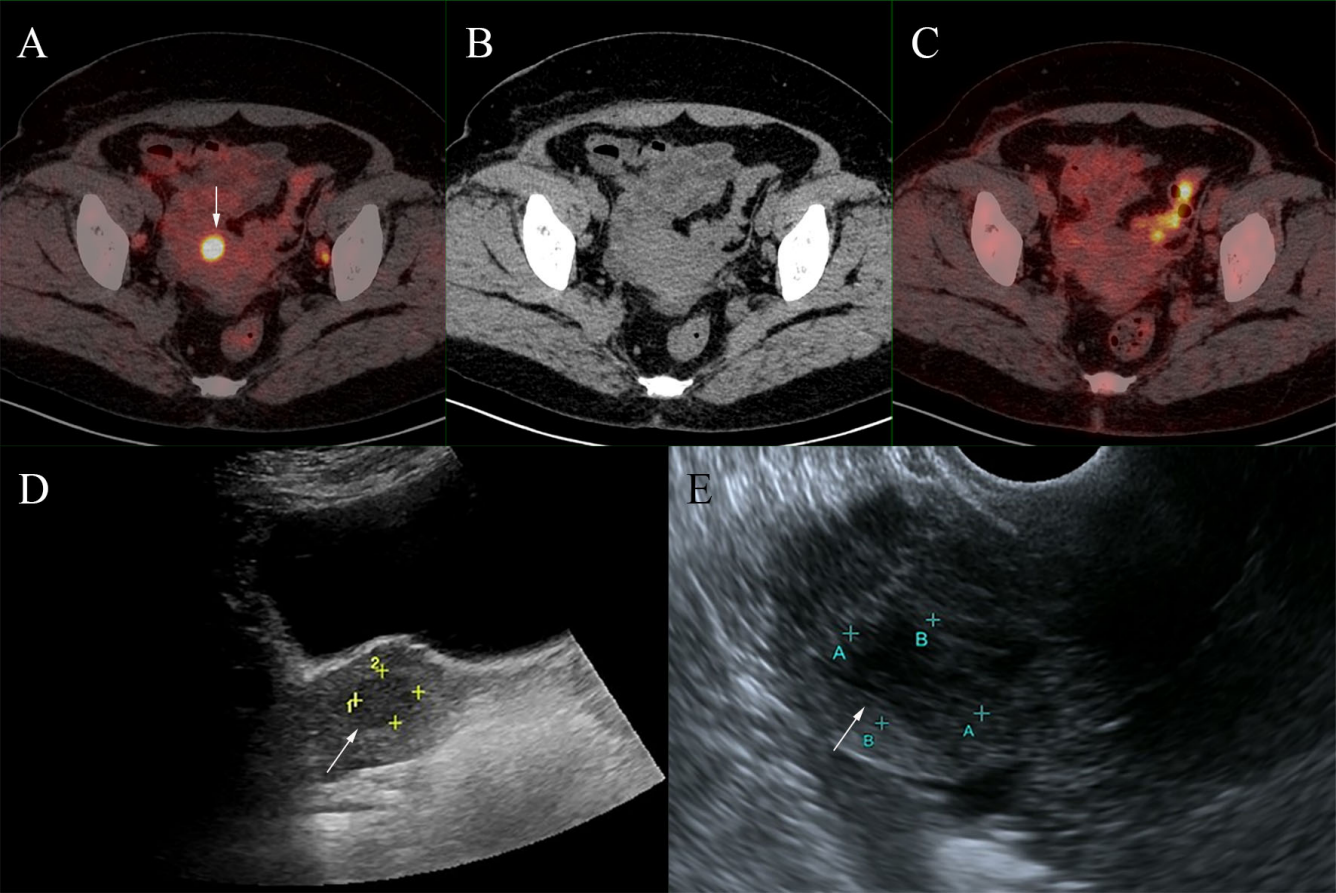
A foci of increased ^68^Ga-DOTATATE activity (**A**, fused PET/CT) was located in the uterus (SUVmax,11.0; white arrows), but neither notable CT structural alterations nor ^18^F-FDG activity was seen (**B**, CT; **C**, fused PET/CT). This lesion was found to be uterine fibroids after ultrasound examination (**D**, transabdominal ultrasonography; **E**, transvaginal ultrasonography).

Then ^18^F-FDG PET/CT was performed for an unknown primary origin (**Figure 1**) and no abnormal activity was observed in the MIP image. The axial images of PET/CT found enlarged lymph nodes at the bilateral axillary and right inguinal with mild FDG avidity and no abnormality was seen in the bilateral breasts. Then the patient underwent ^68^Ga-DOTATATE PET/CT one day after ^18^F-FDG PET/CT (**Figure 1**). In the ^68^Ga-DOTATATE MIP image and the axial images, higher uptake of ^68^Ga-DOTATATE than ^18^F-FDG was observed in the enlarged bilateral axillary and right inguinal lymph nodes, but bilateral breasts were still negative. Moreover, a lesion with increased ^68^Ga-DOTATATE activity was located in the uterus with, but neither notable CT structural alterations nor ^18^F-FDG activity can be seen (**Figure 2**).

**Figure 3:**
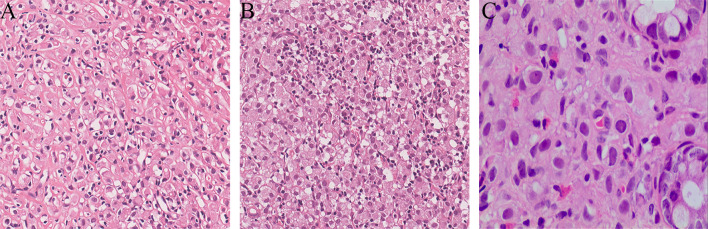
Ultrasound-guided percutaneous biopsy for the ^68^Ga-DOTATATE-avid left axilla and right inguinal lymph nodes. The pathological results revealed metastatic invasive lobular breast carcinoma (**A**, left axillary lymph node, HE×200; **B**, right inguinal lymph node, HE×200). The rectal polyp confirmed metastatic carcinoma of suspected breast origin (**C**, rectum polyp, HE×200).

Due to unknown primary origin, this patient underwent ultrasound-guided percutaneous biopsy for the ^68^Ga-DOTATATE-avid left axilla and right inguinal lymph nodes, and the pathological results revealed metastatic invasive lobular breast carcinoma (**Figure 3**). Interestingly, the histopathology from the left axillary lymph node showed “No lymph node tissue seen”. Finally, the patient was diagnosed with occult breast cancer with metastases to the lymph nodes and rectum.

## Discussion

Occult breast cancer is defined as an axillary metastatic carcinoma without detection of a primary breast lesion in a patient, and without a history of prior breast cancer, without clinical, radiological, or pathological evidence of a primary lesion in the breast. According to previous literature and case reports, the most common sites of pathological diagnosis or metastasis were the axilla, bone, and orbit. Meanwhile, some uncommon sites had also been reported, including gastrointestinal tract, liver, lung, thyroid, and brain (8–10). OBC patients with distant metastatic disease have a much worse prognosis, with a 5-year survival of 14.3% (8). Thus, it is of great significance to ascertain whether there is distant metastasis in patients of OBC.

Several case reports have shown that primary breast cancer can demonstrate avidity to ^68^Ga-DOTA peptides (11–13). Andrew et al. reported that ^68^Ga-DOTATATE was as sensitive as ^18^F-FDG in staging of primary ER+/PR+ breast cancers (8/10 *vs.* 8/10) (14). Another case report showed a primary invasive lobular breast carcinoma lesion with ^68^Ga-DOTATATE activity, which was not ^18^F-FDG-avid (11). About 50% of breast tumors express SSR, ^68^Ga-DOTA peptide PET/CT examination can incidentally detect breast tumors (15–17). However, the report about ^68^Ga-DOTATAE PET/CT detection of occult breast cancer remain rare. In our case, the primary breast cancer remained radiologically insidious, however, bilateral axillary and right inguinal lymph nodes metastases showed significantly increased ^68^Ga-DOTATATE uptake and better tumor-to-background ratio than ^18^F-FDG. Our case suggested that ^68^Ga-DOTATAE PET/CT maybe an effective supplement in diagnosing OBC lymph node metastases with mild ^18^F-FDG uptake.

One previous study suggested that occult breast cancer may originate from ectopic breast tissue presented in axillary lymph nodes, and emphasized that the immunohistochemical subtype in OBC comprised various types similar to primary breast cancer (18). In our case, histopathology from left axillary lymph node metastases showed “No lymph node tissue seen’’, and this may be the origin of OBC. Perhaps ^68^Ga-DOTATAE PET/CT is as useful in the diagnosis of the origin of OBC as it is in the detection of OBC metastatic lesions.

Furthermore, the ^68^Ga-DOTATATE-avid lesion located in the uterus was found to be uterine fibroids by further ultrasound examination, corroborating the finding of a recent case report (19). The false-positive results of ^68^Ga-DOTATATE may lead to diagnostic challenges and require further differentiation.

## Conclusions

Our case report provides preliminary evidence that ^68^Ga-DOTATATE PET/CT may be a helpful clinically problem-solving imaging modality in diagnosing occult breast cancer, especially in diagnosing metastatic lesions.

## Data availability statement

The original contributions presented in the study are included in the article/supplementary material. Further inquiries can be directed to the corresponding authors.

## Ethics statement

Written informed consent was obtained from the individual(s) for the publication of any potentially identifiable images or data included in this article.

## Author contributions

TH, RZ, JH, BW and ZZ: manuscript writing. LL and YZ: pathological review. TH, RZ, BZ, SH, ZZ, and PH: manuscript revision. All authors contributed to the article and approved the submitted version.
